# Effects of Different *Chenopodium formosanum* Parts on Antioxidant Capacity and Optimal Extraction Analysis by Taguchi Method

**DOI:** 10.3390/ma14164679

**Published:** 2021-08-19

**Authors:** Chin-Tung Wu, Wei-Hsun Wang, Wen-Shin Lin, Shiou-Yih Hu, Cheng-You Chen, Min-Yun Chang, Yung-Sheng Lin, Chi-Ping Li

**Affiliations:** 1 Bachelor Program in Interdisciplinary Studies, College of Future, National Yunlin University of Science and Technology, Yunlin 640301, Taiwan; ctwu5@yuntech.edu.tw; 2 Department of Orthopedic Surgery, Changhua Christian Hospital, Changhua 500209, Taiwan; cmch10011@gmail.com; 3 School of Medicine, Kaohsiung Medical University, Kaohsiung 807378, Taiwan; 4 Department of Golden-Ager Industry Management, Chaoyang University of Technology, Taichung 413310, Taiwan; 5 Department of Medical Imaging and Radiology, Shu-Zen Junior College of Medicine and Management, Kaohsiung 821004, Taiwan; 6 College of Medicine, National Chung Hsing University, Taichung 402202, Taiwan; 7 Department of Chemical Engineering, National United University, Miaoli 360001, Taiwan; qaz86890123@gmail.com (S.-Y.H.); mino101451@gmail.com (M.-Y.C.); 8 Department of Plant Industry, National Pingtung University of Science and Technology, Pingtung 912301, Taiwan; wslin@mail.npust.edu.tw; 9 Ph.D. Program in Materials and Chemical Engineering, National United University, Miaoli 360001, Taiwan; wayne20410@gmail.com; 10 Institute of Food Safety and Health Risk Assessment, National Yang Ming Chiao Tung University, Taipei 112304, Taiwan

**Keywords:** antioxidant, *Chenopodium formosanum*, leaf, extract, design of experiment, Taguchi method

## Abstract

*Chenopodium formosanum* (CF), rich in nutrients and antioxidants, is a native plant in Taiwan. During the harvest, the seeds are collected, while the roots, stems, and leaves remain on the field as agricultural waste. In this study, di(phenyl)-(2,4,6-trinitrophenyl)iminoazanium (DPPH) radical scavenging ability and 2,2′-azino-bis(3-ethylbenzthiazoline-6-sulfonic acid) (ABTS) radical scavenging ability experiments of seeds, leaves, stems, and roots were designed using the Taguchi method (TM) under three conditions: Ethanol concentration (0–100%), temperature (25–65 °C), and extraction time (30–150 min). The result demonstrates that seeds and leaves have higher radical scavenging ability than stems and roots. Many studies focused on CF seeds. Therefore, this study selected CF leaves and optimized DPPH, ABTS, total phenol content (TPC), total flavonoid content (TFC), and reducing power (RP) through TM, showing that the predicted value of the leaf is close to the actual value. The optimized results of CF leaves were DPPH 85.22%, ABTS 46.51%, TPC 116.54 µg GAE/mL, TFC 143.46 µg QE/mL, and RP 23.29 µg VCE (vitamin C equivalent)/mL. The DPPH and ABTS of CF leaves were second only to the results of CF seeds. It can be seen that CF leaves have the potential as a source of antioxidants and help in waste reduction.

## 1. Introduction

In recent years, people have paid more and more attention to the ingredients and additives from health foods and cosmetics. The level of efficacy is no longer a priority for consumers. Synthetic antioxidants are rejected by consumers who admire nature. The natural, non-toxic antioxidants extracted from plants with antioxidant properties will become the mainstream in the future. Recent reports show that medicinal plants provide pharmacological and biological activities [1,2,3]. Some plants have anticancer potential [4].

Many studies show that the most nutrient part of a plant may not be its seed, for example, some roots such as the carrot [5,6,7] and the potato [8,9,10]. Some leaves contain essential antioxidants and bioactive compounds such as *Arum maculatum* leaves [11], *Moringa oleifera* L. leaves [12], and *Pluchea indica* L. leaves [13].

In recent years, *Chenopodium formosanum* (CF) has become a popular nutritious food on the market. It is also known as djulis or red quinoa. CF is an important component of Taiwanese biodiversity for Taiwanese diet or health food. Taiwan CF was selected as the main object of this study. CF has quite rich nutrients and antioxidants [14,15]. Its bright red appearance is rich in beet pigment [16]. It has earned the reputation of grain ruby. CF combined with *Lactobacillus acidophilus* shows an inhibitory effect on colon carcinogenesis [14,15]. CF provides insoluble dietary fiber which can postpone the adsorption of glucose and reduce the activity of α-amylase as well as benefit type 2 diabetes mellitus patients [14]. However, when CF is harvested, the farmers usually only harvest the economically valuable CF seeds, and the remaining roots, stems, and leaves are burned as agricultural waste, which is quite wasteful. From the literature [17,18,19,20,21,22,23], it is found that many researches have focused on the efficiency of the CF seeds. To our best knowledge, there are few reports about functional analysis on leaves of CF [22,23]. This may be due to the fact that most scientists believe that seeds consist of more antioxidants and nutrients. However, CF leaves also contain a lot of antioxidants, similar to seeds. After analyzing various parts of CF, the CF leaf was selected for more in-depth research in this article. The authors are the first team looking at the economic value of CF leaves other than CF seeds.

Taiwan CF and quinoa are rich in nutrients, with high protein, dietary fiber, trace elements, and amino acids required by the human body. From the literature [24,25], it can be observed that they have different contents of nutrients and each has its own advantages. CF has a higher proportion of dietary fiber, protein, calcium, iron, magnesium, phosphorus, selenium, vitamins, and folic acid, while quinoa contains higher starch, lipid, potassium, and zinc content, all of which are foods that can meet the basic nutritional needs of humans when taken alone. CF has eight essential amino acids that cannot be synthesized by the human body, including threonine, lysine, valine, methionine, isoleucine, leucine, phenylalanine, and histidine. All of them need to be ingested from food, and have very important effects on the human body [25,26]. 

A design of experiment (DOE) can also be called quality engineering or robust design. Various fields have different ways of addressing it. In engineering, it is mostly called the design of experiment method. Regardless of what it is called, experimental design hopes to meet a logical experiment arrangement, experiment with the least time and cost, and obtain the most suitable parameters and quality characteristics [27]. The Taguchi method (TM) omits the statistical theory and calculation part of the experimental design, and directly analyzes the experimental orthogonal table and experimental data, making it easier to understand and apply in the food industry practice. TM has been used to optimize the process of heavy metal extraction [28], essential oil extraction [29], ultrafiltration [30], drying foods [31,32], and baking parameters [33]. 

In the study of antioxidant capacity, organic solvent extraction is quite common, but it is often studied by trial and error. There are many experiments and it is difficult to find the optimal conditions and results. In this work, three extraction conditions such as ethanol concentration, temperature, and time were selected. TM was used to perform optimal analysis, obtain the best extraction conditions and results, evaluate whether CF leaves have the potential to be a source of natural antioxidants, and achieve the goal of waste reduction in this investigation. This study investigated the conditions to obtain optimal antioxidant capacity such as DPPH, ABTS, TPC, TFC, and RP of CF seeds, leaves, stems, and roots using TM. 

## 2. Materials and Methods 

### 2.1. Reagents

The 2,2’-azino-bis(3-ethylbenzthiazoline-6-sulfonic acid) (ABTS), aluminium(III) chloride, and quercetin were obtained from Alfa Aesar (Tewksbury, MA, USA). Vitamin C was purchased from Acros (Geel, Belgium). Sodium hydroxide was provided by Choneye Chemical Co. (Taipei, Taiwan). Ethanol was procured from Echo Chemical Co. (Miaoli, Taiwan). Potassium ferricyanide was purchased from First Chemical Co. (Pascagoula, MS, USA). Folin-Ciocâlteu’s (Folin) reagent was obtained from Fisher Scientific (Loughborough, Leicestershire, UK). Gallic acid (GA) was provided by Fluka (Neu-Ulm, Germany). Iron(III) chloride hexahydrate was purchased from J.T. Baker (Phillipsburg, NJ, USA). Sodium carbonate, sodium phosphate dibasic dihydrate, and trichloroacetic acid were obtained from Riedel-de Haën (Seelze, Germany). Sodium phosphate monobasic was provided by Shimakyu’s Pure Chemical Co. (Osaka, Japan). Potassium perdisulfate was procured from Showa Chemical Co. (Tokyo, Japan). Di(phenyl)-(2,4,6-trinitrophenyl)iminoazanium and sodium nitrite were purchased from Sigma-Aldrich (St. Louis, MO, USA).

### 2.2. Extraction of Chenopodium Formosanum

*Chenopodium formosanum* was collected from the Liang Sin Protection Farm, Yunlin, Taiwan. The whole CF plant was divided into four parts: Seed, root, stem, and leaf, cut into pieces and placed in a hot-air circulating oven (30 °C, 72 h) to remove water. After that, the sample was fully dried, crushed, and measured by a sieve for the particle size. This study selected the powder that passes mesh 30 and does not pass mesh 80. The average particle size was 383.5 μm. All of the processed samples were stored in the freezer (−30 °C) for later use. 

When extracting various parts of CF, the ratio of 1 g powder to 100 mL ethanol solution was fixed, and the powder and ethanol solution were placed in a frosted conical flask. The bottle mouth was sealed tightly with a frosted glass stopper. Then, the flasks were placed in a reciprocating oscillating water tank with constant temperature for extraction. The extract was obtained by suction filtration, and placed in a freezer (−30 °C) for storage.

In this study, ethanol concentration (%), temperature (°C), and time (min) were selected as the control factors. After multiple tests and empirical judgments, the range of each factor was set. The range of ethanol concentration was set 5~95%. If the temperature was more than 70 °C during extraction, the closed Erlenmeyer flask cannot withstand the pressure of ethanol volatilization, causing the cork to pop up or the bottle body to crack, affecting the progress of the study, so the temperature was set 25–65 °C. The time was set in the range of 30~150 min based on previous experimental data.

### 2.3. Antioxidant Capacity Analysis

#### 2.3.1. DPPH Radical Scavenging Assay

In the research of antioxidant capacity, DPPH radical scavenging ability is often used to test the hydrogen supply capacity of antioxidants. DPPH is a kind of stable free radical. The experimental procedure was in accordance with the literature [34,35,36,37,38] with a slight modification. In addition, 2 mL of freshly prepared 0.08 mg/mL DPPH ethanol solution was added into 2 mL of CF extract, mixed evenly, and kept in the dark for 30 min. The absorbance at a wavelength of 517 nm was measured, and compared with the blank control group to calculate the free radical scavenging ability. The calculation equation is as follows:

(1) $$\mathrm{DPPH}\;\mathrm{radical}\;\mathrm{scavenging}\;\mathrm{ability}\left(\%\right)\;=\;\left(1-\frac{\mathrm{Sample}\;\mathrm{absorbance}}{\mathrm{Blank}\;\mathrm{control}\;\mathrm{group}\;\mathrm{absorbance}}\right)\times 100\%$$

#### 2.3.2. ABTS Radical Scavenging Assay

ABTS reacts with the strong oxidizing agent, potassium persulfate, to generate stable ABTS^+^ cationic radicals, which are blue-green with a high absorption peak at a wavelength of 734 nm. The experimental procedure was in accordance with the literature [35,37,39] with a slight modification. Then, 5 mL of 7 mM ABTS was mixed with 5 mL of 2.45 mM K_2_S_2_O_8_, reacted for 16 h in the dark, and then diluted with 95% ethanol to make the absorbance at 0.7 ± 0.05. Following that, 3.6 mL of the prepared ABTS ethanol solution was added into 0.4 mL of the CF extract, mixed evenly, and stood in the dark for 10 min. The absorbance at a wavelength of 734 nm was measured and compared with the blank control group by a 0.4 mL ethanol solution replacing a 0.4 mL CF extract to calculate the removal capacity as follows:

(2) $$\mathrm{ABTS}\;\mathrm{radical}\;\mathrm{scavenging}\;\mathrm{ability}\left(\%\right)\;=\;\left(1-\frac{\mathrm{Sample}\;\mathrm{absorbance}}{\mathrm{Blank}\;\mathrm{control}\;\mathrm{group}\;\mathrm{absorbance}}\right)\times 100\%$$

#### 2.3.3. Determination of Total Phenol Content

The experimental procedure refers to the method of the literature [40] with slight modifications. Additionally, 1.5 mL of 1 N Folin and 1.2 mL of 15% Na_2_CO_3_ were added into 0.3 mL of CF extract, mixed well, and stood for 30 min in the dark. Then, the absorbance value at a wavelength of 765 nm was measured, calculated by the GA calibration curve, and expressed as the amount of GA per gram of dry extract (mg GAE/g DW).

#### 2.3.4. Determination of Total Flavonoid Content

Flavonoids are natural phenolic compounds which are widely found in plants. They are the most diverse branch of polyphenols. The experimental procedure was in accordance with the literature [16] with a slight modification. Additionally, 0.15 mL of 5% NaNO_2_ was added into 1 mL of CF extract, and stood for 5 min in the dark, then 0.3 mL of 10% AlCl_3_ was added, and stood in the dark for 5 min. After that, 1.5 mL of 1 M NaOH was added at the end, mixed evenly, and then stood for 30 min in the dark. The absorbance at a wavelength of 510 nm was measured and calculated with the quercetin calibration curve. The expression is the equivalent of the dry weight of each gram of quercetin extract (mg QE/g DW).

#### 2.3.5. Determination of Reducing Power

The reduction ability measurement mainly uses the amount of ferricyanide (Prussian blue) produced as an index of the reduction ability. The experimental procedure was in accordance with the literature [41] with a slight modification. Additionally, 45 mL of 0.2 M Na_2_HPO_4_ and 75 mL of 0.2 M NaH_2_PO_4_ were mixed uniformly to prepare a phosphate buffer solution (2 mM, pH 6.6). Then, 0.5 mL of 2 mM buffer solution and 0.5 mL of 1% K_3_Fe(CN)_6_ were added into 1 mL of CF extract, mixed evenly, and placed in a water bath at 50 °C in the dark for 20 min, and then fully cooled in an ice bath. After cooling, 0.5 mL of 10% CCl_3_COOH, 2.5 mL of DI water, and 0.5 mL of 0.1% FeCl_3_ were added, mixed evenly and stood for 10 min in the dark. The absorbance at a wavelength of 700 nm was measured and calculated by the vitamin C calibration curve. The expression is how much vitamin C (mg VCE/g DW) is equivalent to the dry weight of each gram of extract.

### 2.4. Taguchi Method 

The L_9_(3^3^) orthogonal array was selected based on three factors, including the ethanol concentration (%), extraction temperature (°C), and extraction time (min). Three levels of each factor were selected for TM as shown in Table 1. The Taguchi method transfers the quality characteristics into an S/N ratio to evaluate the statistical values of the performance. The selected quality characteristic of the antioxidant activity was the larger-the-better S/N ratio [42]. The S/N the ratio is a measure of robustness used to identify control factors that reduce variability in a product or process by minimizing the effects of uncontrollable factors (noise factors). The S/N ratio is as follows:

(3) $$S/N=10\;{\;\log}_{10}\left(\frac{\mathrm{signal}}{\mathrm{noise}}\right)$$

### 2.5. Statistical Analysis

To ensure the statistical differences between the treatments, the replication of treatment is 3, and means between the treatment levels were compared using the SAS software (version 9.4, 2016, SAS Institute, Cary, NC, USA). Statistical analyses are based on the analysis of variance (ANOVA). When the ANOVA test gets a significant result (*p* < 0.05), Fisher’s protected least significant difference (LSD) test is used to compare the different ability among each part of CF. The Pearson correlation coefficient is estimated and evaluated with the correlation procedure (PROC CORR) in SAS. 

## 3. Results

### 3.1. Using the Taguchi Method to Analyze the Antioxidant Capacity of Various Parts of CF

Table 1 is the Taguchi method factor. The level selection for analyzing the antioxidant capacity of each part of CF, the selection of extraction condition level, and ethanol concentration (A) are 25, 50, 75%, temperature (B) are 30, 40, 50 °C, and time (C) are 45, 60, 75 min.

The Trolox, a well-known antioxidant standard, was used as the positive control for DPPH and ABTS radical scavenging assay, and the determined 50% inhibitory concentration of Trolox for DPPH and ABTS were both less than 10 μg/mL, validating these assays in this study. Table 2 shows the DPPH and ABTS radical scavenging activity of each part of CF. The DPPH radical scavenging activity of the leaf was higher than the seed. Then, the seed was higher than the stem, and the root was the lowest. The ABTS radical scavenging activity had a similar situation similar to DPPH. 

After the calculation, the S/N ratio of each part of CF can be obtained as shown in Table 3. From Table 3, the ethanol concentration, temperature, and extraction time all had a big influence on the seed and leaf for both DPPH and ABTS. The ethanol concentration had great influence on the stem of DPPH. 

The optimal conditions and results were predicted by Table 4. The predicted value of seed for DPPH and ABTS was higher than the leaf. The optimized conditions of DPPH were 50% ethanol concentration and 50 °C for both the seed and leaf. The optimized conditions of ABTS were 25% ethanol concentration for the seed, stem, and leaf. It seems that the root required higher ethanol concentration (75%) to obtain an optimal extraction.

### 3.2. Using the Taguchi Method to Analyze the Antioxidant Capacity of Leaves

Table 5 is the Taguchi Orthogonal Table for optimizing the extraction of CF leaves. The selection of extraction conditions was compared with Table 1 which analyzes the antioxidant capacity of each part of CF, and the range of temperature and time were increased. The ethanol concentrations (A) are 25, 50, 75%, temperature (B) are 25, 45, 65 °C, and time (C) are 30, 90, 150 min.

Table 6 is a summary of the results of optimizing the antioxidant capacity of CF leaves. It can be seen that the best results of CF leaf antioxidant capacity were DPPH 85.22%, ABTS 46.51%, TPC 116.54 μg GAE/mL, and TFC 154.32 μg QE/mL, RP was 23.29 μg VCE/mL. The predicted value and the actual value had a slight gap but were not large. Furthermore, the predicted value and the actual value had a highly positive correlation (r = 0.99, *p* < 0.0001).

## 4. Discussion

### 4.1. Using the Taguchi Method to Analyze the Antioxidant Capacity of Various Parts of CF

From Table 2, most of the DPPH radical scavenging activity of the leaf was more than 80% over the three control factors, and this indicated that the leaf of CF contains lots of antioxidants. This ABTS radical scavenging activity was the same as DPPH. The leaf was higher than the seed. The rank of the DPPH and ABTS radical scavenging activity was leaf > seed > stem > root.

The S/N ratio of DPPH from Table 3 shows that the ethanol concentration had a significant effect on seeds, stems, and leaves. The time had a greater influence on the root of DPPH. It also indicated that the ethanol concentration had a major effect on seeds, roots, and stems from the S/N ratio of ABTS in Table 3. Time had a greater influence on leaves, and the optimal conditions and results were predicted in Table 4. 

Table 4 shows the optimal conditions and results for the DPPH and ABTS radical scavenging ability of each part of CF. The optimal extraction conditions for seeds and leaves were 50% ethanol concentration and 50 °C temperature. The time was 60 min and 75 min, respectively. Seeds had the best DPPH scavenging ability, and the second was leaf, but there was little difference between the two abilities. The best results of DPPH of seeds and leaves were 96.23 and 89.47%, respectively. The best extraction conditions for roots and stems were ethanol concentration 75 and 25%, temperature 50 and 30 °C, and the time was 60 and 75 min. This result was relatively poor compared with seed and leaf, and exhibited almost no scavenging ability. The DPPH radical scavenging ability of seeds and leaves were much higher than the report of previous research of seeds such as 65.68 [19] and 47.88% [18]. The DPPH value of leaves was a little lower than seeds but much higher than stems and roots, and showed the second best DPPH radical scavenging ability other than seeds. In each column of Table 4, there are no significant differences according to the result of the LSD test (*p* < 0.05).

The scavenging ability of ABTS radicals demonstrated that the optimal extraction conditions of each part of CF were different. The best ethanol concentration of seeds, stems, and leaves were all 25%, and the best ABTS scavenging ability was seed, the second was leaf, but there was little difference between the abilities of these two. The best results of ABTS of seeds and leaves were 70.48 and 55.85%, respectively. The results of roots and stems were relatively poor, and there were almost no scavenging abilities. The ABTS values of seeds and leaves were lower than the previous study of CF seeds after fermentation (93.8%) [43].

After testing, it can be known that the ABTS radical scavenging ability of each part of CF was ranked by seeds, leaves, stems, and roots. The CF leaves demonstrated second best ABTS radical scavenging ability to the seeds. There have been quite a lot of researches on antioxidants of CF seeds [18,19,43,44], but there is relatively few researches on CF leaves. Therefore, the Taguchi method was used to optimize the antioxidant capacity of CF leaves for more in-depth research. 

### 4.2. Using the Taguchi Method to Analyze the Antioxidant Capacity of Leaves

Table 5 is the Taguchi Orthogonal Table modified to obtain the optimal extraction conditions of CF leaves. The extraction temperature was extended from 30, 40, 50 °C to 25, 45, 65 °C. The extraction time was also extended from 45, 60, 75 min to 30, 90, 150 min. The reason for the increase in the range of extraction temperature and time was to avoid the possibility of local optimum.

From Table 6, the DPPH value of CF leaves was higher than the whole grain seeds from other journal such as 65.68 [19] and 47.88% [18]. The TPC value of leaves was 97.19 mg GAE/g DW, which was higher than the reports of seeds (26.11 mg GAE/g DW [19], 3.24 mg GAE/g DW [44], and 804.67 mg GAE/100 g DW [18]). The TFC value of leaves was 156.15 mg QE/g DW, which was much higher than the article of seeds (2.55 mg QE/g DW [44] and 350 mg QE/100 g DW [18]). The RP value of leaves was 23.29 μg VCE/mL, which was higher than the reports of *Hypogymnia physodes* 7.97 μg VCE/mL [45]. The native Taiwan CF seeds and leaves contain a much higher amount of antioxidants than the CF seeds from the other place [18].

The result was approved by the previous study [18,19,44], and the CF grain contained an abundance of polyphenols. The previous article [46] provided evidence that grains with red and black pericarp colors obtained higher amount of total phenolic content, total flavonoid content, and antioxidant capacity, and that there were significant positive correlations between those variables. Moreover, one research [44] found a high positive correlation between the total phenolic content and total flavonoid content (r = 0.91, *p* < 0.0001). These flavonoid compounds provide regulation of oxygen generation, involved in free radical formation, scavenging reactive compounds, and protecting antioxidants [47,48]. One literature [18] that studied the high pressure process treatment of CF hulls exhibited the highest total flavonoid content (910.27–1011.73 mg QE/100 g). A previous study [14,49] revealed that CF contains a high quantity of phenolic and flavonoid compounds that display the potential of its use in the development of enriched food products due to its ability to lower LDL-cholesterol and high free radical scavenging capacities.

This research provided good evidence of the CF leaf as a potential candidate to acquire the antioxidants other than CF seeds. Hopefully, the leaf of CF can be used as a source of antioxidants and minimization of agricultural waste.

## 5. Conclusions

In this study, the Taguchi method was applied to optimize the extraction of different CF parts such as seeds, leaves, stems, and roots, which greatly avoided various problems caused by trial and error methods, such as minimizing research costs, reducing the number and time of experiments, and obtaining scientifically and systematically optimal extraction conditions and antioxidant capacity. The Taguchi method pointed out that the seeds and leaves of CF had stronger antioxidant capacity compared with roots and stems of CF. The DPPH and ABTS radical scavenging ability of seeds and leaves were much higher than that of the roots and stems. Moreover, the radical scavenging ability of leaves is second only to seeds. The predictive value of DPPH, ABTS, TPC, TFC, and RP of leaves from TM are close to the results of the actual value. In the future, it is expected that CF leaves can exert its potential and achieve the goal of the CF waste recycling.

## Figures and Tables

**Table 1:** Taguchi method factors and level selection for analyzing the antioxidant ability of each part of *Chenopodium* *formosanum*.

Factor	Coded	Factor Level		
		1	2	3
Ethanol concentration (%)	A	25	50	75
Extraction temperature (°C)	B	30	40	50
Extraction time (min)	C	45	60	75

**Table 2:** The DPPH and ABTS radical scavenging ability of each part of *Chenopodium formosanum*.

L_9_(3^3^)	Factor				DPPH Radical Scavenging Activity (%)					ABTS Radical Scavenging Activity (%)			
	A	B	C		Seed	Root	Stem	Leaf		Seed	Root	Stem	Leaf
1	1	1	1		48.47 ± 1.87	3.35 ± 0.50	11.16 ± 0.27	78.42 ± 0.71		41.85 ± 2.84	3.05 ± 0.84	2.50 ± 1.30	47.52 ± 1.69
2	1	2	2		78.59 ± 1.68	3.66 ± 0.69	15.48 ± 0.69	80.54 ± 0.64		52.87 ± 1.54	2.61 ± 0.45	6.89 ± 1.02	51.39 ± 1.27
3	1	3	3		87.59 ± 0.23	5.20 ± 1.12	10.99 ± 0.59	81.60 ± 0.48		62.79 ± 2.64	1.00 ± 0.38	4.94 ± 0.92	51.68 ± 0.69
4	2	1	2		59.14 ± 2.31	4.82 ± 1.15	12.45 ± 1.35	80.12 ± 0.87		47.23 ± 3.28	2.19 ± 1.31	10.52 ± 1.25	48.35 ± 1.13
5	2	2	3		85.02 ± 0.38	1.30 ± 0.44	8.95 ± 0.56	80.49 ± 0.98		53.04 ± 0.78	1.68 ± 0.29	6.22 ± 1.16	50.88 ± 1.14
6	2	3	1		81.50 ± 0.63	1.45 ± 0.50	14.93 ± 1.11	61.58 ± 1.64		51.64 ± 1.08	3.64 ± 1.40	8.12 ± 1.65	46.74 ± 1.62
7	3	1	3		26.39 ± 1.42	4.29 ± 0.71	12.98 ± 0.32	82.83 ± 1.03		20.60 ± 2.04	6.85 ± 1.04	5.82 ± 1.29	52.32 ± 1.03
8	3	2	1		40.33 ± 1.52	2.68 ± 0.74	5.76 ± 0.34	84.08 ± 0.39		27.83 ± 3.98	2.81 ± 0.70	4.46 ± 0.68	48.20 ± 0.99
9	3	3	2		67.87 ± 1.39	6.55 ± 1.14	4.60 ± 0.53	83.13 ± 0.58		41.55 ± 2.33	6.06 ± 0.61	5.87 ± 0.25	46.27 ± 1.35

**Table 3:** The S/N ratio of each part of *Chenopodium formosanum*.

Part	Level	S/N Ratio						
		DPPH				ABTS		
		A	B	C		A	B	C
seed	1	36.82	33.74	34.67		34.26	31.51	31.78
	2	37.41	36.20	36.65		34.06	32.55	33.42
	3	32.38	37.90	35.28		29.08	34.18	32.21
	range	5.03	4.16	1.98		5.18	2.67	1.63
	rank	1	2	3		1	2	3
root	1	11.73	10.37	6.66		5.16	6.80	8.96
	2	5.39	6.63	13.37		5.62	6.92	8.90
	3	12.06	10.64	9.15		13.50	7.88	6.42
	range	6.67	4.01	6.70		8.34	1.08	2.53
	rank	2	3	1		1	3	2
stem	1	21.84	19.31	19.85		12.04	13.04	12.20
	2	21.41	19.32	19.57		17.92	15.00	17.42
	3	16.85	19.12	20.69		14.32	15.56	14.65
	range	4.99	0.20	1.12		5.88	2.52	5.22
	rank	1	3	2		1	3	2
leaf	1	38.08	38.15	37.39		34.00	33.62	33.52
	2	37.32	38.24	38.20		33.73	34.00	33.73
	3	38.42	37.47	38.24		33.78	33.65	34.25
	range	1.09	0.77	0.85		0.27	0.38	0.73
	rank	1	3	2		3	2	1

**Table 4:** The summary of antioxidant capacity and conditions of each part.

Part	DPPH					ABTS			
	Optimization (A, B, C)	Predictive Value	Actual Value	Error (%)		Optimization (A, B, C)	Predictive Value	Actual Value	Error (%)
seed	50, 50, 60	94.98	96.23 ± 0.58 ^a^*	1.30		25, 50, 60	62.96	70.48 ± 0.95 ^a^	10.67
root	75, 50, 60	6.27	8.59 ± 0.51 ^d^	27.01		75, 30, 45	5.80	8.54 ± 0.87 ^d^	32.17
stem	25, 30, 75	14.10	18.44 ± 1.01 ^c^	23.55		25, 50, 60	10.06	11.49 ± 0.67 ^c^	12.47
leaf	50, 50, 75	88.29	89.47 ± 1.00 ^b^	1.32		25, 40, 75	53.46	55.85 ± 1.31 ^b^	4.29

* Means with the same lowercase letters in the same column are not significant differences according to the result of the LSD test (*p* < 0.05).

**Table 5:** Taguchi method factors and level selection for analyzing the antioxidant capacity of leaves of *Chenopodium* *formosanum*.

Factor	Coded	Factor Level		
		1	2	3
Ethanol concentration (%)	A	25	50	75
Extraction temperature (°C)	B	25	45	65
Extraction time (min)	C	30	90	150

**Table 6:** The results of optimization of *Chenopodium formosanum* leaf antioxidant capacity and conditions by the Taguchi method.

Part	Optium Conditions (A, B, C)	Predictive Value	Actual Value	Error (%)
DPPH (%)	50, 25, 90	83.64 ± 0.63	85.22	1.88
ABTS (%)	75, 25, 30	48.21 ± 0.94	46.51	3.53
TPC (μg GAE/mL)	50, 45, 90	122.00 ± 0.90	116.54	4.48
TFC (μg QE/mL)	75, 65, 90	154.32 ± 3.83	143.46	7.04
RP (μg VCE/mL)	25, 45, 90	24.75 ± 0.16	23.29	5.90

## Data Availability

All the data is available within the manuscript.
